# Comparison of new forms of creatine in raising plasma creatine levels

**DOI:** 10.1186/1550-2783-4-17

**Published:** 2007-11-12

**Authors:** Ralf Jäger, Roger C Harris, Martin Purpura, Marc Francaux

**Affiliations:** 1 Increnovo LLC, 2138 E Lafayette Pl, Milwaukee, WI 53202, USA; 2 School of Sport, Exercise and Health Sciences, University College Chichester, College Lane, Chichester, West Sussex, PO19 4PE, UK; 3 Institut d'Education physique et de Readaptation, Universite catholique de Louvain, Louvain-la-Neuve, Belgium

## Abstract

**Background:**

Previous research has shown that plasma creatine levels are influenced by extracellular concentrations of insulin and glucose as well as by the intracellular creatine concentration. However, the form of creatine administered does not appear to have any effect although specific data on this is lacking. This study examined whether the administration of three different forms of creatine had different effects on plasma creatine concentrations and pharmacokinetics.

**Methods:**

Six healthy subjects (three female and three male subjects) participated in the study. Each subject was assigned to ingest a single dose of isomolar amounts of creatine (4.4 g) in the form of creatine monohydrate (CrM), tri-creatine citrate (CrC), or creatine pyruvate (CrPyr) using a balanced cross-over design. Plasma concentration curves, determined over eight hours after ingestion, were subject to pharmacokinetic analysis and primary derived data were analyzed by repeated measures ANOVA.

**Results:**

Mean peak concentrations and area under the curve (AUC) were significantly higher with CrPyr (17 and 14%, respectively) in comparison to CrM and CrC. Mean peak concentration and AUC were not significantly different between CrM and CrC. Despite the higher peak concentration with CrPyr there was no difference between the estimated velocity constants of absorption (ka) or elimination (kel) between the three treatments. There was no effect of treatment with CrPyr on the plasma pyruvate concentration.

**Conclusion:**

The findings suggest that different forms of creatine result in slightly altered kinetics of plasma creatine absorption following ingestion of isomolar (with respect to creatine) doses of CrM, CrC and CrPyr although differences in ka could not be detected due to the small number of blood samples taken during the absorption phase. Characteristically this resulted in higher plasma concentrations of creatine with CrPyr. Differences in bioavailability are thought to be unlikely since absorption of CrM is already close to 100%. The small differences in kinetics are unlikely to have any effect on muscle creatine elevation during periods of creatine loading.

## Background

Creatine supplementation has been reported to increase muscle creatine and phosphorylcreatine content by 5 to 30% [1]. However, significant intra-subject variability has been reported in regards to the magnitude that creatine stores and performance are affected [1-3]. Initial evidence reported by Chanutin [4] and recently confirmed by Deldicque et al. [5] indicates that intestinal absorption of creatine supplied as the monohydrate is close to 100%.

The aim of this pilot study was to compare the effects of ingesting isomolar amounts of creatine (4.4 g) in the form of the monohydrate (5 g), tri-creatine citrate (6.7 g) and creatine pyruvate (7.3 g) on creatine concentrations in plasma. The study was prompted by the fact that salts of creatine are more quickly taken up into solution than the monohydrate, and thus could offer advantages over the monohydrate. The inclusion of the small amounts of pyruvate and citrate were thought unlikely to influence creatine absorption and uptake, although initial studies with 25 g pyruvate plus 75 g dihydroxyacetone have indicated an ergogenic effect of pyruvate supplementation through a proposed stimulation of glucose uptake by muscle [6]. However, no blood samples were taken in that study to measure plasma pyruvate levels during the period of oral supplementation [6,7]. Smaller doses of calcium pyruvate [6–10 g/d], on the other hand, showed mixed results in clinical trials [8-10]. Acute calcium pyruvate supplementation of 7 g, 15 g or 25 g did not result in any significant elevation of blood pyruvate levels [11] and consumption of 7 g/d for 7 days did not result in improved aerobic endurance in well-trained athletes [11,12].

## Methods

### Subjects

Three females and three males participated in this pilot study. All subjects in this investigation participated in a familiarization session. During the familiarization session, subjects were informed as to the experimental procedures, completed a personal/medical history form, creatine supplementation history form and signed informed consent statements in adherence with the human subject's guidelines of the American College of Sports Medicine. The study was approved by the Ethical Review Committee of the University of Chichester. Subject characteristics are presented in table 1. No subject in this trial was a vegetarian with all subjects reportedly consuming meat in their daily diet.

**Table 1:** Subjects characteristics

**No.**	**Sex**	**Age (years)**	**Weight (kg)**	**Height (m)**
1	M	21	84	1.82
2	M	29	87	1.82
3	M	22	78	1.86
4	F	55	70	1.58
5	F	51	65	1.62
6	F	35	68	1.65

### Supplementation Protocol

The study used a cross-over design. Each subject received the three treatments using a latin-square design on three different days, with 7 days allowed between each treatment. Treatments comprised:

A: 5 g Creatine monohydrate (CrM) dissolved in 450 ml of water (control)

B: 6.7 g Tri-creatine citrate (CrC) dissolved in 450 ml of water (isomolar with respect to creatine to A and C)

C: 7.3 g Creatine pyruvate (CrPyr) dissolved in 450 ml of water (isomolar with respect to creatine to A and B).

The creatine monohydrate (Creapure™) contains 88% w/w creatine, creatine pyruvate (Creapure™Pyruvate) contains 60% w/w creatine and 40% w/w pyruvate, and, tri-creatine citrate (Creapure™Citrate) contains 65% w/w creatine. All three were obtained from Degussa AG (Trostberg, Germany) and contained <100 ppm creatinine, whilst dicyandiamide and dihyrdotriazine levels and polymeric pyruvates in CrPyr were not detectable by HPLC.

### Procedures

Two ml blood samples were collected into tubes containing lithium heparin through an antecubital vein, centrifuged and plasma was harvested and stored frozen until analysed. Plasma (500 μl) was extracted with 15 μl of 70% w/w PCA and supernatants after centrifugation were neutralised with a minimum volume of 2N KHCO_3_at 4°C. One hundred and sixty one blood samples, from an intended 162, were collected. Data for from the missing sample (subject 1, treatment C, 2 hours) were estimated from the two adjacent points assuming an exponential decrease between 1.5 to 3 hours. Creatine in plasma extracts was analysed photometrically by following the oxidation of NADH at 340 nm in the presence of creatine kinase, pyruvate kinase and lactate dehydrogenase [13]. Pyruvate was analysed using a similar assay but with the addition of lactate dehydrogenase only.

### Pharmacokinetics

Using a Marquardt algorithm, all kinetics were fitted with the following equation:

C = (FD/V*(ka/(ka-kel)) * (exp(-kel*t) - exp(-ka*t)))

Where:

C is the plasma creatine concentration minus the basal concentration (μM)

if C ≤ 0, then data were removed

F is the bioavailability

D is the dose (5 g or 38.17 mmol)

V is the volume of distribution

ka is the velocity constant of absorption (first order, h^-1^)

kel is the velocity constant of elimination (first order, h^-1^)

t is the time (h)

No weight was applied to data (weight = 1) and the initial parameters values were estimated using a Simplex algorithm. This equation corresponds to a one-open compartmental model. The area under the curve (AUC) from zero to infinity was estimated by non compartmental model

### Statistical Analysis

Results are shown as means together with standard deviation. Primary and derived variables were analysed by repeated measures ANOVA. Where a significant effect of treatment was indicated, data were further compared using a Bonferroni post-hoc test. The threshold for significance was set at p < 0.05.

## Results

**Figure 1:**
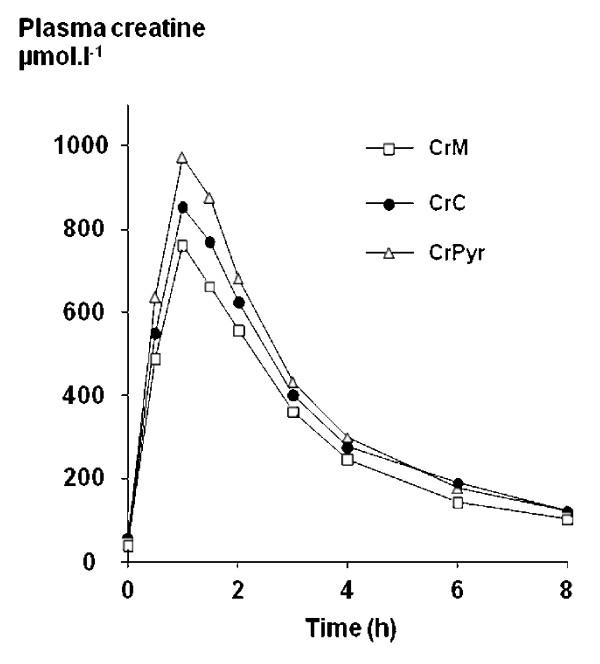
Mean plasma creatine concentration over 8 hours following ingestion of 4.4 g of Creatine in the form of creatine monohydrate (CrM), tri-creatine citrate (CrC), or creatine pyruvate (CrPyr). Dispersions and statistical significances are given in table 2.

Mean (SD) plasma concentrations following the three treatments are shown in table 2 and the mean curves are depicted in figure 1. One hour after the ingestion, the plasma creatine concentration was higher with CrPyr than with CrC (p = 0.024, table 2). The difference between means was larger when CrPyr and CrM were compared, but in this last case the signification threshold was not reached (p = 0.089).

**Table 2:** Mean (SD) plasma creatine concentration over 8 hours following ingestion of isomolar amounts of creatine (4.4 g) in the form of creatine monohydrate (CrM), tri-creatine citrate (CrC), or creatine pyruvate (CrPyr). Means within each time-row differing significantly are denoted by the letters a to c.

**Time (h)**	**CrM (a)**		**CrC (b)**		**CrPyr (c)**		**ANOVA P value**
		
	**Mean**	**SD**	**Mean**	**SD**	**Mean**	**SD**	
0	40.5	25.5	56.5	36.7	44	28	NS
0.5	488.6	185.7	551.1	191.3	637	207.2	NS
1	761.9	107.7	855.3 c	165.1	972.2 a	184.1	0.015
1.5	660.8	159.2	771.8	289.3	875.7	310.9	0.012
2	557	162.1	624.2	263.1	681.7	299.4	NS
3	362.5	174.4	400.1	235.7	431.5	228.5	NS
4	247.8	134.8	276	169.3	298	176.3	NS
6	142.7	87.3	189.1	120.2	178.5	110.3	NS
8	103.4	64.5	123.1	83.6	122.9	83.7	NS

Peak concentrations were always the highest after ingestion of CrPyr. Mean peak concentrations were 1.17 fold higher with CrPyr compared to CrC (p = 0.010) and 1.29 fold higher than with CrM, but again missing the threshold of significance (p = 0.075, table 3). The time at which the peak concentrations were reached (T_max_) was unaffected by the form of creatine ingested as well as the velocity constants of absorption (ka) and elimination (ke) (table 3). The mean half-life of elimination was 30 ± 11.6 min. AUC values with CrPyr were also the highest in all subjects. Mean AUC with CrPyr was 1.14 fold higher compared to CrC although the difference did not reach the threshold of significance (P = 0.069).

**Table 3:** Mean (SD) peak plasma concentration (Cr_max_, mM), time to peak concentration (T_max_, h), area under the plasma concentration curve (AUC, mM.h), velocity constant of absorption (ka, h^-1^) and velocity constant of elimination (ke, h^-1^) following ingestion of isomolar amounts of creatine (4.4 g) in the form of creatine monohydrate (CrM), tri-creatine citrate (CrC), or creatine pyruvate (CrPyr). Means differing significantly are denoted by the letters a to c.

	**CrM (a)**		**CrC (b)**		**CrPyr (c)**		**ANOVA P value**
		
	**Mean**	**SD**	**Mean**	**SD**	**Mean**	**SD**	
**Cr**_max_	751	32.9	837 c	71.5	968 a	82.3	0.008
**T**_max_	1.5 c	0.13	1.2	0.1	1.1 a	0.08	0.002
**AUC**	2384	376.5	2627	506.8	2985	540.6	0.023
**ka**	1.26	0.214	1.39	0.551	1.31	0.324	NS
**kel**	0.73	0.344	0.66	0.279	0.72	0.279	NS

**Figure 2:**
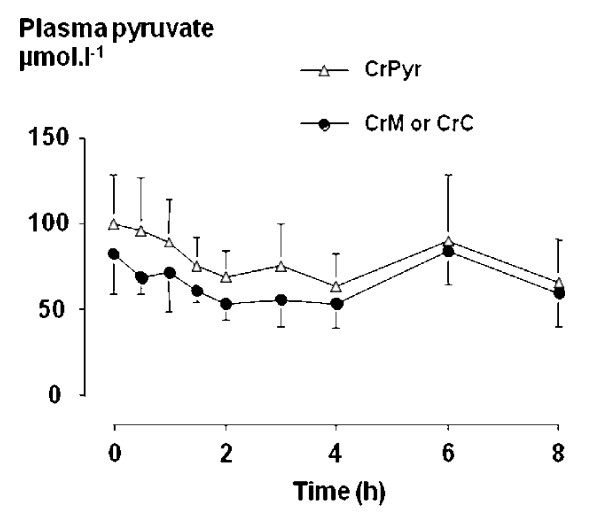
Mean plasma pyruvate concentrations during 8 hours following ingestion of creatine pyruvate (CrPyr, 7.3 g), creatine monohydrate (CrM, 5 g) or tri-creatine citrate (CrC, 6.7 g).

There was no effect of treatments on the plasma pyruvate concentrations (figure 2).

## Discussion

Although creatine is widely used as a dietary supplement, only a few studies describe data relevant to its pharmacokinetics [1,5,14-19]. After ingestion of a single low dose, the time-course of the changes in plasma concentration is generally accepted to follow a one-open compartment model with first-order absorption and elimination constants [20]. The present results support this model.

All treatments at all doses resulted in substantial increases in plasma creatine which if repeated could be expected to lead to enhancement of the creatine content in muscle [1,2]. However, some differences were apparent in the dynamics of plasma creatine following ingestion of isomolar (with respect to creatine) doses of CrM, CrC and CrPyr. Characteristically this resulted in higher plasma concentrations of creatine with CrPyr. This may suggest that faster absorption or slower elimination of creatine when supplied as CrPyr although this was not evident from the estimates of ka and kel. Faster absorption of creatine could, for instance occur, as a result of an increase in metabolisable substrate (i.e. pyruvate or citrate) for use by enterocytes. It is likely that the small change in ka was missing due to the small number of blood samples taken during the absorption phase, namely 3–4 samples. Higher plasma concentrations could also indicate suppression of uptake by muscle, possibly through the lowering of insulin in the circulation or due to changes in plasma pH as a result of the metabolism of pyruvate and citrate supplied, but this is not supported by the kel values which were calculated over a larger number of data points. The higher plasma concentration with CrPyr is unlikely to have been due to greater bioavailability, since the bioavailability of CrM is known to be close to 100% [4,5]. Differences in muscular creatine levels during and after supplementation would be needed to allow a difference in muscular performance. It is questionable if the small differences observed in the plasma concentrations with the three treatments would have any effect on the increase in creatine in muscle. However, this would need to be confirmed by direct measurement.

The inability to detect any change in the plasma pyruvate concentration is attributed to the rapid clearance and metabolism of this by the liver; although decarboxylation in the stomach and intestine and/or elimination through the urine or faeces [10] are also possibilities.

## Conclusion

The findings suggest that different forms of creatine result in slightly altered kinetics of plasma creatine absorption following ingestion of isomolar (with respect to creatine) doses of CrM, CrC and CrPyr although differences in ka could not be detected due to the small number of blood samples taken during the absorption phase. Characteristically this resulted in higher plasma concentrations of creatine with CrPyr. Differences in bioavailability are thought to be unlikely since absorption of CrM is already close to 100%.

## Competing interests

The author(s) declare that they have no competing interests.

## Authors' contributions

RH, RJ and MP participated in the design of the study. RH organized the blood collection and assayed the samples, and, together with MF analyzed the results statistically. MF performed the pharmacokinetic analysis of the data, and, RJ, RH and MF drafted the manuscript. All authors have read and approved the final manuscript.
